# Supplementary material for: Inflammatory factors and risk of lung adenocarcinoma: a Mendelian randomization study mediated by blood metabolites
Source: Front Endocrinol (Lausanne). 2024 Aug 27;15:1446863. doi: 10.3389/fendo.2024.1446863 (PMC11384989; doi:10.3389/fendo.2024.1446863)
Supplement: Supplementary file 1 [file DataSheet1.zip › supplementary files/S2/GCST90200549/forest.pdf]

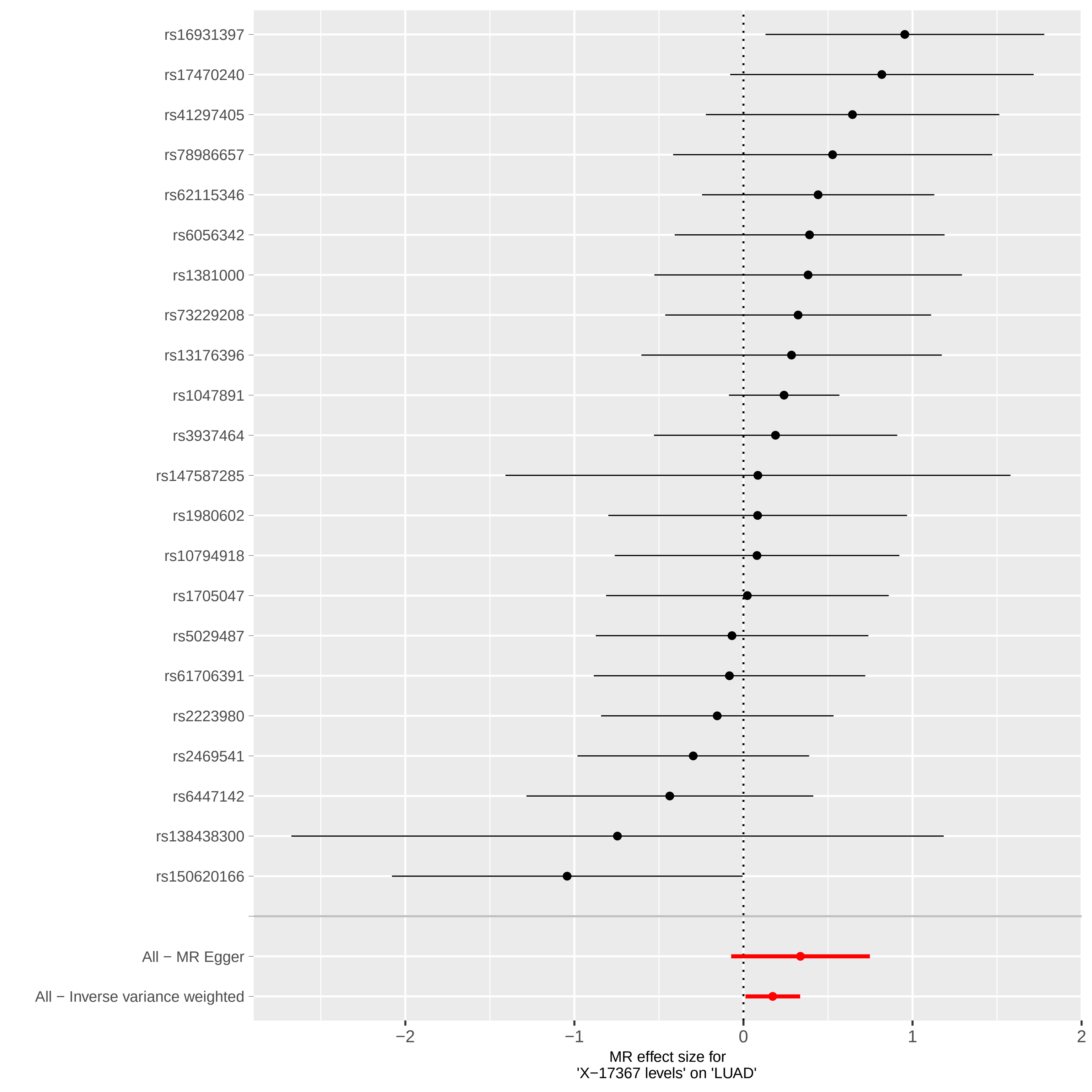

rs16931397
rs17470240
rs41297405
rs78986657
rs62115346
rs6056342
rs1381000
rs73229208
rs13176396
rs1047891
rs3937464
rs147587285
rs1980602
rs10794918
rs1705047
rs5029487
rs61706391
rs2223980
rs2469541
rs6447142
rs138438300
rs150620166
All − MR Egger
All − Inverse variance weighted
−2
−1
0
1
2
MR effect size for
'X−17367 levels' on 'LUAD'
